# The complete mitochondrial genome of Chinese minnow (*Rhynchocypris oxycephalus*) and its phylogenetic analyses

**DOI:** 10.1080/23802359.2021.1875921

**Published:** 2021-07-05

**Authors:** Cheng Zhang, Shun Zhang, Zhe Tian, Shun Cheng, Danli Wang, Shanliang Xu

**Affiliations:** a School of Marine Science, Ningbo University, Ningbo, China; b Key Laboratory of Applied Marine Biotechnology, Ningbo University, Chinese Ministry of Education, Ningbo, China

**Keywords:** *Rhynchocypris oxycephalus*, Phylogenetic relationship, Mitochondrial genome

## Abstract

The complete mitochondrial genome can provide novel insights into understanding the mechanism underlying mitogenome evolution. In the present study, the whole mitochondrial genome of *Rhynchocypris oxycephalus* was determined to 16608 bp (GenBank accession No: MW057563), including 13 protein-coding genes, 22 transfer RNA genes, two ribosomal RNA genes, and one control region. The overall base composition was 28.62% A, 27.23% T, 26.31% C and 17.84% G, with a total A + T content of 55.85%. The Maximum Likelihood tree showed that the phylogenetic relationship is closer between *R. oxycephalus* and *Phoxinus oxycephalus jouyi* than the other species. The whole mitogenome of this species will be useful for the future animal evolutionary, phylogenetic relationship, and genomic studies in the genus *Phoxinus*.

Chinese minnow (*Rhynchocypris oxycephalus*) is a small cold-water fish, which is endemic to East Asia and generally inhabits stream headwaters (Jang et al. 2002; Bogutskaya et al. 2008). The life history characteristics of *R. oxycephalus* (such as low reproductive output, short life cycle, and low growth rate) and its ecological tolerance characteristics determine that *R. oxycephalus* is extremely sensitive and vulnerable to environmental changes or human interference, and their local populations are extremely vulnerable (Yu et al. 2013; Chu et al. 2015). Therefore, it is necessary to raise concern about long-term conservation of *R. oxycephalus*. Little is however known about the conservation status of *R. oxycephalus*. The classification status of this species and the phylogenetic relationship of genus *Phoxinus* was very complicated (Ito et al. 2002; Sasaki et al. 2007). To gain a better insight into its taxonomic relationship, the specimen was collected from Yongjiang River (121.63°E, 29.90°N), Ningbo City, Zhejiang Province, China, and they were deposited in Key Laboratory of Applied Marine Biotechnology, Ningbo University (Sample code is YG-150422). The entire genomic DNA was extracted from 30 to 50 mg of muscle tissue using the standard phenol-chloroform extracting method (Sambrook and Russell 2001), and preserved at −20 °C. Based on the conserved sequences of *P. semotilus* and *P. oxycephalus jouyi* (Miya et al. 2015; Yu et al. 2017), we designed 18 pairs of primers for polymerase chain reaction amplification. The PCR fragments were assembled by BioEdit version 7.2.5 software (Hall 1999) and then calculated the nucleotide base composition by MEGA6.0 (Tamura et al. 2013). To avoid assembling error, the complete mitochondrial sequence was aligned with its closely related species by BLAST. The annotated genomic sequence has been submitted to GenBank under the accession number MW057563.

In total, the complete mtDNA of *R. oxycephalus* was 16608 bp in length, and the content was consistent with the typical fishes’ mitochondrial genomes (Perna and Kocher 1995). It contained 13 PCGs, 22 tRNA genes, two rRNA genes, and one control region. Among the 37 genes, one PCGs (*ND6*) and eight tRNA genes (*tRNA^Gln^*, *tRNA^Ala^*, *tRNA^Asn^*, *tRNA^Cys^*, *tRNA^Tyr^*, *tRNA^Ser1(UGC)^*, *tRNA^Glu^*, *tRNA^Pro^*) were on the light strand, and the remaining 28 genes were on the heavy strand. In 13 protein-coding genes, apart from *COI* utilizing GTG, the rest of the 12 protein-coding genes start with the same initiation codon ATG. The typical termination codons (TAA or TAG) were detected in 10 PCGs (TAA for *ND1*, *COI*, *ATP6*, *COX III*, *ND4L*, and *ND5* genes, TAG for *ATP8*, *ND3*, *ND4*, and *ND6* genes), and the remaining three genes (*ND2*, *COII*, and *Cyt b*) were ended by incomplete stop codons (T–). The length of all tRNAs ranged from 68 to 76 bp. The *O_L_* region (L-strand replication origin) was located between *tRNA^Asn^* and *tRNA^Cys^*. The *12S* and *16S rRNA* genes are 954 bp and 1670 bp, respectively. The control region of 935 bp was located in *tRNA^Pro^* and *tRNA^Phe ^*(Table 1).

**Table 1.** Mitogenome characteristic of ***Rhynchocypris*** *oxycephalus*.

Gene/element	Position		Length (bp)	Codon		Anticodon	Strand^a^
	From	To		Start codon	Stop codon		
*tRNA^Phe^*	1	69	69			GAA	H
*12S rRNA*	70	1023	954				H
*tRNA^Val^*	1026	1097	72			TAC	H
*16S rRNA*	1117	2786	1670				H
*tRNA^Leu1(UAA)^*	2788	2863	76			TAA	H
*ND1*	2865	3839	975	ATG	TAA		H
*tRNA^Ile^*	3844	3915	72			GAT	H
*tRNA^Gln^*	3914	3984	71			TTG	L
*tRNA^Met^*	3986	4054	69			CAT	H
*ND2*	4055	5101	1046	ATG	T —		H
*tRNA^Trp^*	5100	5170	71			TCA	H
*tRNA^Ala^*	5172	5240	69			TGC	L
*tRNA^Asn^*	5242	5314	73			GTT	L
*O_L_*	5318	5347	30				–
*tRNA^Cys^*	5348	5415	68			GCA	L
*tRNA^Tyr^*	5417	5487	71			GTA	L
*COI*	5489	7039	1551	GTG	TAA		H
*tRNA^Ser1(UGC)^*	7040	7110	71			TGA	L
*tRNA^Asp^*	7114	7187	74			GTC	H
*COII*	7201	7891	691	ATG	T—		H
*tRNA^Lys^*	7892	7967	76			TTT	H
*ATPase8*	7969	8133	165	ATG	TAG		H
*ATPase6*	8127	8810	684	ATG	TAA		H
*COIII*	8810	9594	785	ATG	TAA		H
*tRNA^Gly^*	9594	9664	71			TCC	H
*ND3*	9665	10015	350	ATG	TAG		H
*tRNA^Arg^*	10014	10082	69			TCG	H
*ND4L*	10083	10379	297	ATG	TAA		H
*ND4*	10373	11751	1379	ATG	TAG		H
*tRNA^His^*	11755	11823	69			GTG	H
*tRNA^Ser2(GCU)^*	11824	11891	68			GCT	H
*tRNA^Leu2(UAG)^*	11893	11965	73			TAG	H
*ND5*	11966	13801	1836	ATG	TAA		H
*ND6*	13798	14319	522	ATG	TAG		L
*tRNA^Glu^*	14320	14388	69			TTC	L
*Cyt b*	14391	15531	1141	ATG	T—		H
*tRNA^Thr^*	15532	15603	72			TGT	H
*tRNA^Pro^*	15603	15673	71			TGG	L
*D-loop*	15674	16608	935				–

^a^H and L indicate heavy and light strands, respectively.

To investigate the phylogenetic relationship among the genus *Phoxinus*, the mitochondrial genome sequences of seven currently available species of *Phoxinus* were downloaded, including *P. oxycephalus jouyi* (AP011269.1), *P. semotilus* (NC_029341.1), *P. steindachneri* (NC_015357.1), *P. keumkang* (AP011363.1), *P. tumensis* (KC992395.1), *P. phoxinus* (AB671170.1), and *P. ujmonensis* (NC_023802.1), together with *Acrocheilus alutaceus* (NC_033927.1) as outgroup species. The phylogenetic tree was constructed using Maximum Likelihood method based on complete mtDNA. Tree topology was evaluated by 1000 bootstrap replicates, and the tree had high bootstrap supporting values. The result indicated that the phylogenetic relationship is closer between *R. oxycephalus* and *P. oxycephalus jouyi* than the other species (Figure 1).

**Figure 1.**
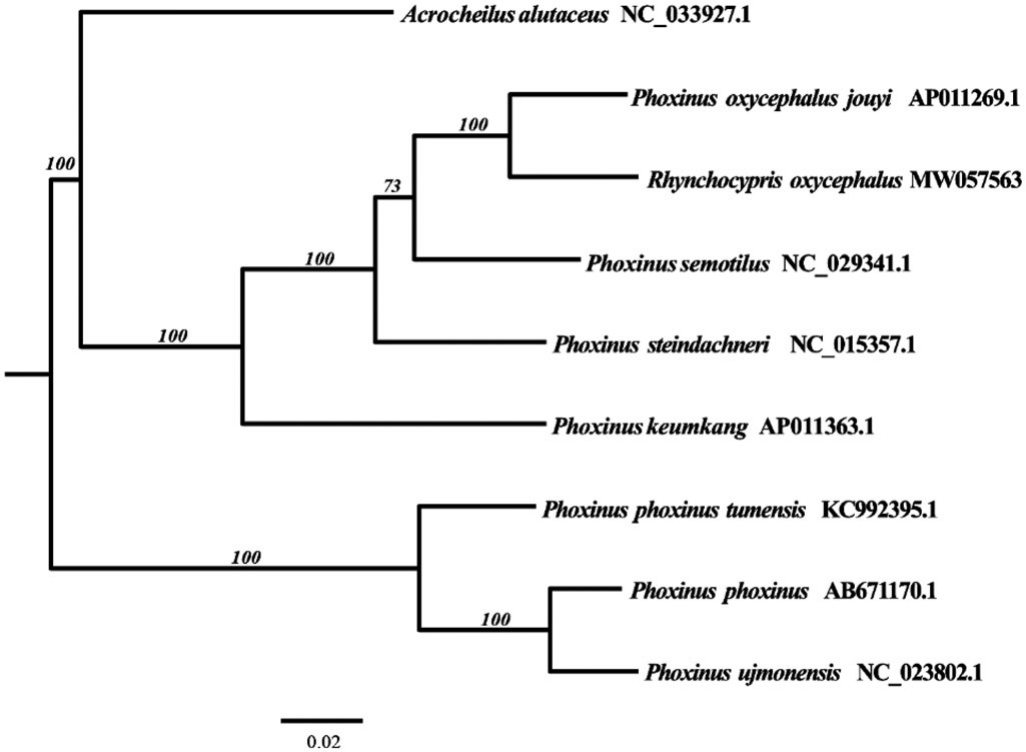
The Maximum Likelihood tree inferred from the complete mitogenomes of nine species.

## Data Availability

The data that support the findings of this study are available in GenBank of NCBI at https://www.ncbi.nlm.nih.gov, reference number MW057563.
